# Association between leukocyte telomere length and sex by quantile regression analysis

**DOI:** 10.1016/j.htct.2020.12.005

**Published:** 2021-02-06

**Authors:** Fernanda Gutierrez-Rodrigues, Raquel M. Alves-Paiva, Natália F. Scatena, Edson Z. Martinez, Priscila S. Scheucher, Rodrigo T. Calado

**Affiliations:** a Faculdade de Medicina de Ribeirão Preto da Universidade de São Paulo (FMRP-USP), Ribeirão Preto, SP, Brazil; b Hospital Israelita Albert Einstein Hospital, São Paulo, SP, Brazil

**Keywords:** Quantile regression, Sex, Telomere length, Flow-fish

## Abstract

**Introduction:**

Telomere length (TL) is a biomarker of cellular proliferative history. In healthy individuals, leukocyte TL shortens with age and associates with the lifespan of men and women. However, most of studies had used linear regression models to address the association of the TL attrition, aging and sex.

**Methods:**

We evaluated the association between the TL, aging and sex in a cohort of 180 healthy subjects by quantile regression. The TL of nucleated blood cells was measured by fluorescent *in situ* hypridization (flow-FISH) in a cohort of 89 men, 81 women, and 10 umbilical cord samples. The results were validated by quantitative polymerase chain reaction (qPCR) and compared to a linear regression analysis.

**Results:**

By quantile regression, telomere dynamics slightly differed between sexes with aging: women had longer telomeres at birth and slower attrition rate than men until the sixth decade of life; after that, TL eroded faster and became shorter than that in men. These differences were not observed by linear regression analysis, as the overall telomere attrition rates in women and men were similar (42 pb per year, *p* < 0.0001 *vs*. 45 pb kb per year, *p* < 0.0001). Also, qPCR did not recapitulate flow-FISH findings, as the telomere dynamics by qPCR followed a linear model.

**Conclusion:**

The quantile regression analysis accurately reproduced a third-order polynomial TL attrition rate in both women and men, but it depended on the technique applied to measure TL. The Flow-FISH reproduced the expected telomere dynamics through life and, differently from the qPCR, was able to detect the subtle TL variations associated with sex and aging.

## Introduction

Telomeres are DNA-protein structures located at the ends of linear chromosomes that prevent aberrant activation of DNA-damage responses and genome instability.^1^ During cellular replication, the DNA polymerase is intrinsically unable to fully duplicate telomeres and, although telomerase is expressed in highly proliferative cells, telomeres invariably get shorter with aging.^2^ The telomere length (TL) dynamics inversely correlates with age, but the association with sex is still controversial, as some studies found longer telomeres in women,[3], [4], [5] whereas others found no differences.^6^

Most studies comparing the leukocyte TL between men and women estimate the statistical parameters by the least squares method from linear regression models, with a corresponding standard error and regression coefficients. However, telomere dynamics follows a third-order polynomial model, rather than a linear one; telomere attrition is faster in early childhood, followed by a plateau until the sixth decade of life, then rapidly shortening again.[2], [7], [8], [9] Quantile regression, also known as the least-absolute value regression, is an appropriate tool to estimate the parameters in polynomial models, as no distribution assumption is required and it is robust against outliers.[10], [11], [12] Whereas the least squares method is used to quantify the relationship between a dependent variable and some covariates based on average, the quantile regression method uses the median or other quantiles to display curves that better describe these relationships.

To investigate the contribution of the quantile regression analysis to the study of the telomere dynamics between the sexes, we measured the TL of peripheral blood leukocytes collected from healthy men and women by the flow-FISH. Results were further compared linear regression analysis.

## Methods

### Healthy individuals

For this study, ethylenediamine tetraacetic acid (EDTA) blood samples were collected from 180 healthy volunteers (men, n = 89; women, n = 81), ranging from zero to 92 years, and umbilical cord blood (n = 10) at the Hospital das Clínicas da Faculdade de Medicina de Ribeirão Preto da Universidade de São Paulo (HC-FMRP-USP). For comparison analysis, an independent cohort of 242 healthy individuals in which TL was measured by a different technique was also included in the study. The local Ethics Committee approved the study (process number, 12050/2011) and written consent was obtained from all participants or their legal guardians before sample collection.

### Telomere length measurement

In the two independent cohorts, TL was measured by either the fluorescence *in situ* hybridization and flow cytometry (flow-FISH; n = 180) or quantitative polymerase chain reaction (qPCR; n = 242), as previously described.[7], [9], [13] Measurements were analyzed according to the individuals’ age and sex.

### Statistical analysis

For quantile regression, scatter plot graphics were used to assist in the visualization of the best pattern that could describe the relationship between the age and TL before adjusting the model. These graphs suggested that a third order polynomial model, given by the following equation, better described the data obtained by flow-FISH: *Y_p_* = *a* + *bx* + *cx*^2^ + *dx*^3^, where the independent variable *x* is the age, Yp is the percentile of the dependent variable telomere length (kb), and *a, b, c* and *d* are the parameters estimated by the quantile regression method.^11^ By this approach, the 1st, 50th (median) and 99th percentiles were simultaneously adjusted to the data and the standard errors for the coefficients were estimated by the bootstrap method. We also fitted a model that includes the sex as a covariate. This model includes interaction terms, allowing for comparisons of the behavior of the curve between men and women. Linear regression was used to calculate the cohort telomere attrition rate with aging and parameters were estimated by the least-squares method. The statistical modelling was performed using the Stata software (v11) and the R software was used to draw the graphs.

## Results

**Fig. 1:**
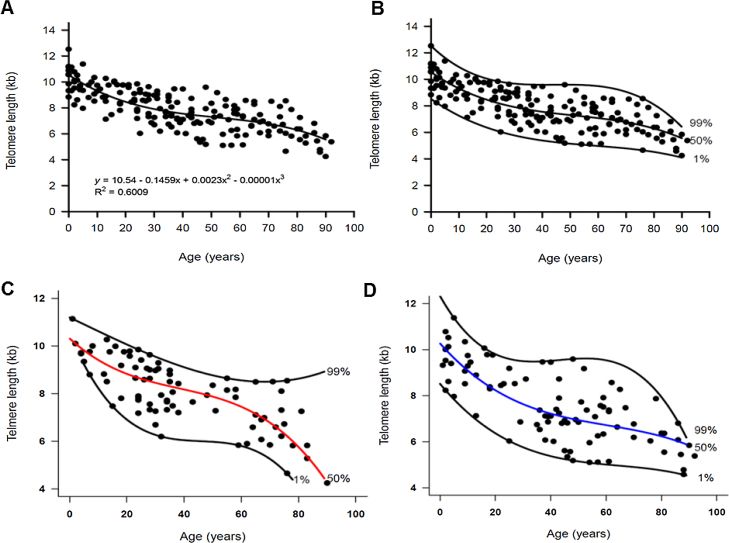
Dynamics of telomere length in women and men described by quantile regression analysis. Telomere length as a marker of aging in the white blood cells of healthy individuals ranging from zero (umbilical cord samples) to 92 years old (n = 180). The vertical axis represents the telomere length in kilobases and the horizontal axis, the age in years. The 1st, 50th (median) and 99th percentiles were adjusted to the data and are shown in the graphics. (A) The relationship between telomere length and age followed a third order polynomial model, described by the equation Yp = a + bx + cx^2^ + dx^3^, shown in the graphic. The independent variable *x* is the age, Yp is the 50th percentile of the dependent variable telomere length (kb) and *a, b, c* and *d* are parameters estimated by the quantile regression. (B) The adjustment of the 1st, 50th (median) and 99th percentiles to the data was performed by the quantile regression. The telomere dynamics through life in (C) women (n = 81) and (D) men (n = 89).

We measured the TL of 89 men (median age, 43 years; range, 1–92), 81 women (median age, 32; range, 1–90), and 10 umbilical cord blood samples by flow-FISH. The TL shortened with aging in both men and women and fitted a third-order polynomial model when using the quantile regression analysis (Fig. 1A–D). In the entire cohort, the telomere attrition rate was 145 bp/year in the first two decades of life, followed by a plateau at 49 bp/year between ages 20–50 years. Erosion rate increased to 62 bp/year after the fifties (Fig. 1A–B and Table 1).

**Table 1:** Telomere attrition in nucleated blood cells of 180 healthy individuals by quantile regression.

	Median TL at birth (kb)	First two decades	Middle age	Old age
		Rate of loss (bcoefficient)	Rate of loss (c coefficient)	Rate of loss (d coefficient)
All (n = 180)	10.5; 95%CI, 10–11	145 bp/year; 95%CI, 213–78 (−0.145x; 95%CI, −0.213, −0.078)	49 bp/year; 95%CI, 63–22 (0.0024x^2^; 95%CI, 0.004, 0.0005)	62 bp/year; 95%CI, 73–33 (−1.54e-05x^3^; 95%CI, −2.9e-05, −1.29e-06)
Women (n = 81)	10.3; 95%CI, 9.4–11.2	109 bp/year; 95%CI, 221–1 (−0.109x; 95% CI, −0.221, 0.0013)	46 bp/year; 95%CI, 73-33 (0.0021x^2^; 95% CI, −0.0011, 0.005)	65 bp/year; 95%CI, 81–51 (−1.87e-05x^3^; 95% CI, −4.45e-05, 7.08e-06)
Men (n = 89)	10.2; 95%CI, 9.2–11.2	135 bp/year; 95%CI, 237–33 (-0.135x; 95% CI, −0.237, −0.033)	43 bp/year; 95%CI, 63–27 (0.002x^2^; 95% CI, −0.0007, 0.004)	56 bp/year; 95%CI, 73–53 (−1.05e-05x^3^; 95% CI, −2.94e-05, 8.37e-06)

The of telomere attrition is not constant with aging, but followed a third polynomial model described by the equation: $y=a+b{x+cx}^{2}+dx^{3}$, where y is the percentile of the variable TL, x is the variable age and a, b, c, and d are the estimated parameters for the 50th percentile. The coefficient a represents the median TL at birth and the coefficients b, c, and d, the different telomere attrition rate through life. The telomere length measurements were performed by the flow-FISH.

**Fig. 2:**
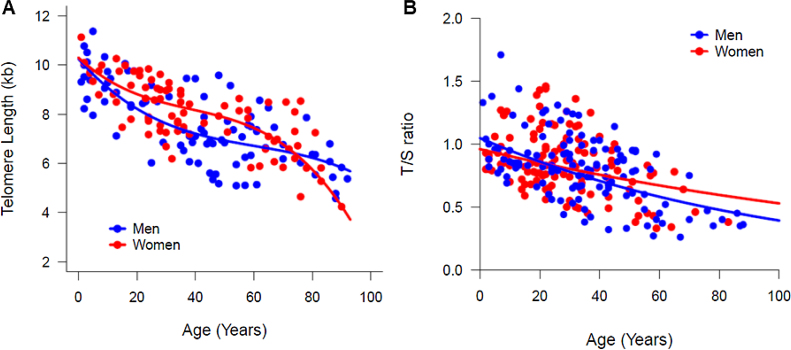
Telomere length (TL) dynamics of according to aging in women (red curve) and men (blue curve) by flow-FISH and qPCR. The comparison of the TL medians (50th percentiles) according to sex. (A) The TL was measured in the white blood cells of 180 healthy individuals, ranging in age from zero (umbilical cord samples) to 92 years old, by the flow-FISH. Women appeared to have longer telomeres and a slower rate of attrition than men. From the fifties on, the telomere length in women decreased rapidly, remaining lower than that of men after the eighties. (B) The TL was measured in white blood cells of 241 healthy individuals, ranging in age from zero (umbilical cord samples) to 88 years old, by the qPCR. No differences in the TL or telomere dynamics was seen between women and men.

In a multiple regression model, by testing the significance of all coefficients concerning the interaction between sex and age, no differences were suggested in the median (50th percentile) TL between women and men. However, a graphical visualization of the percentiles estimated by quantile regression suggested a sex-specific telomere dynamic (Fig. 1C–D). In both women and men, the TL at birth was similar (median TL, 10.26 kb and 10.3 kb in men and women, respectively; Table 1). During the first two decades of life, the telomere attrition in men was slightly faster than in women, and women had longer telomeres until age of 50. Most differences in the TL were seen in older individuals; the telomere attrition rate in women was more pronounced after the age of 60 years, when the TL in women became shorter than that in men (Table 1 and Fig. 2A). This pattern was method-specific and was only observed when TL was measured by flow-FISH (Fig. 2A). When telomeres were measured by qPCR, telomere dynamics by quantile regression were similar to a linear model, misleadingly showing telomere attrition as constant through life (Fig. 2B).

We next compared the analysis using the traditional linear regression model. Similar to the quantile regression, the TL shortening was comparable between sexes; although the average telomere attrition was slower in women than in men, the difference was not statistically significant (42 bp per year *vs*. 45 bp per year, *p* >  0.05; Table 2). The mean TL also was comparable between sexes (women, 8.0 kb ± 1.3; 95 %CI, 7.7–8.3 *vs*. men, 7.3 ± 1.6; 95 % CI, 7.2–8.3; *t* = 1.8, *p* = 0.06). For the entire cohort, the telomere attrition rate was 47 bp/year (95%CI, 53−41; Table 2).

**Table 2:** Telomere attrition in nucleated blood cells of 180 healthy individuals by linear regression.

Age range	n	Linear regression analysis	
		TL at birth (coeficient *a*)	Rate of loss in bp/year (coeficient *b*)
0−92 years	180	9.76 ± 0.14	47 (CI, 53−41)
Women	81	9.70 ± 0.19	42 (CI, 51−34)
Men	89	9.47 ± 0.22	45 (CI, 54−36)
1−20 years	41	9.86 ± 0.26	52 (CI, 100−3)
Women	17	10.6 ± 0.36	194 (CI, 366−19)
Men	24	9.74 ± 0.34	41 (CI, 112−29)
>21 years	129	9.2 ± 0.25	37 (CI, 47−28)
Women	64	9.54 ± 0.29	39 (CI, 51−28)
Men	65	8.5 ± 0.42	30 (CI, 45−15)

The telomere length was measured by the flow-FISH. Linear regression equation: y =a+bx, where parameters a represents the Y- intercept and b, the slope. TL, telomere length; CI, confidence interval; The average TL at birth is given by the intercept and the rate of loss by the slope of the equation that described the data.

When the TL was stratified by age range for linear modeling, the overall attrition rate was 52 bp/year (95%CI, 100−3) in the first two decades of life and 37 bp/year (95%CI, 47−28) in mid-life (> 20 years old; Table 2). When stratified by sex, the TL attrition rate in women younger than 20 years was significantly higher (194 bp/year; 95%CI, 366−19) than in men (41 bp/year; 95%CI, 112−29; *t* test, *p* = 0000.7). However, statistical power may have been limited by the number of women at age < 20 years. Of note, the linear regression did not show any difference in the TL attrition rate according to the technique used for measurement; by flow-FISH and qPCR, the attrition rate was 47 bp/year (95%CI, 53−41) and 40 bp/year (95%CI, 48−33), respectively.

## Discussion

In the present study, the quantile regression analysis was used to investigate whether TL attrition dynamics was different between sexes. Visually, we found that the TL measured by the flow-FISH seems to follow a different pattern of erosion between women and men, although the median TL was not statistically different between the sexes. The quantitative PCR failed to demonstrate the same difference in the erosion pattern between the sexes. Therefore, future studies considering a larger sample size can provide us with better statistical power to compare between men and women.

Several previous studies argued that at birth the TL is similar between the sexes, but with aging, women have longer telomeres than men and their annual shortening rate is lower.[3], [4], [5], [6], [14], [15], [16], [17] A systematic review and meta-analysis corroborate these sex-specific differences in the telomere attrition.^18^ However, the size of these differences varied with the method used to measure the TL. No TL differences between the sexes were found in studies that did not use Southern blot as the measurement approach. Intrinsical methodological differences and technical variability are known to be critical for accurate TL measurement; flow-FISH is comparable to the Southern blot, the gold standard, but is more accurate and precise than the qPCR.^9^ Indeed, in our analysis, flow-FISH but not qPCR was capable of detecting the differences in the TL dynamics associated with aging and sex.

Most importantly, the quantile regression recapitulated the expected age-associated dynamics of TL measured by flow-FISH in both women and men. Adjustment of the 50th percentile (median) showed that the telomere attrition was sex- and age-dependent and may be modulated by physiological events. Until the age of 50 years, women tended to have longer telomeres and a slower attrition rate than men. After that age, the LTL shortened rapidly in women, with a higher attrition rate, when compared to men.

One explanation for the differences in the TL dynamics between women and men is the effects of sex hormones and reactive oxygen species (ROS) on telomere attrition. Estrogens are able to directly activate the telomerase gene promoter and stimulates the addition of catalytic telomere repeats at the end of chromosomes and nitric oxide production. Altogether, estrogens have been linked to telomerase activation and protection against telomere attrition and cellular senescence caused by ROS.[4], [15], [16] Interestingly, androgens have a similar effect on telomerase[16], [17] and could be one reasonable cause for the gradual maintenance of TL in elderly men. Supporting this theory, the age range in which telomere length decreases in women coincides with menopause. It was described that post-menopausal women who had hormone replacement treatment have longer TL than those who had never taken estrogen after menopause.^18^ In our study, sex-specific biological differences influenced telomere dynamics over years. However, we did not have access to the women’s hormone profile and menopause status to comprehensively assesses the relationship between hormones and the TL.

In summary, we found no statistical differences in the median TL between sex of healthy individuals, regardless of the methods used for statistical analysis (quantile or linear regression) or for TL measurement (Flow-FISH or qPCR). However, the telomere attrition dynamics changed according to the technique used to measure the TL. By flow-FISH, women appeared to have longer telomeres and a slower rate of telomere attrition than men until the mid-fifties. After this, the TL decreased rapidly, becoming lower than that of men with the aging. Thus, adjustment of percentiles, instead of the estimation of statistical parameters with a model affected by outliers, was more sensitive to detect physiologic age-dependent differences in telomere dynamics between women and men.

## Conflicts of interest

The authors declare no conflicts of interest.
